# Supplementary material for: T cell activation and deficits in T regulatory cells are associated with major depressive disorder and severity of depression
Source: Sci Rep. 2024 May 16;14:11177. doi: 10.1038/s41598-024-61865-y (PMC11096341; doi:10.1038/s41598-024-61865-y)
Supplement: Supplementary file 1 — Supplementary Figures. [file 41598_2024_61865_MOESM1_ESM.pdf]

**ELECTRONIC SUPPLEMENTARY FILE (ESF1)**

# T cell activation and deficits in T regulatory cells are associated with major depressive disorder and severity of depression

(1) Muanpetch Rachayon; (1,2) Ketsupar Jirakran; (3) Pimpayao Sodsai; (1) Atapol Sughondhabirom, (1,4-7) Michael Maes.

(1) Department of Psychiatry, Faculty of Medicine, Chulalongkorn University and King Chulalongkorn Memorial Hospital, the Thai Red Cross Society, Bangkok, Thailand

(2) Center of Excellence for Maximizing Children's Developmental Potential, Department of Pediatrics, Faculty of Medicine, Chulalongkorn University, Bangkok, Thailand

(3) Center of Excellence in Immunology and Immune-Mediated Diseases, Department of Immunology, Faculty of Medicine, Chulalongkorn University and King Chulalongkorn Memorial Hospital, Bangkok, Thailand

(4) Sichuan Provincial Center for Mental Health, Sichuan Provincial People's Hospital, School of Medicine, University of Electronic Science and Technology of China, Chengdu 610072, China

(5) Key Laboratory of Psychosomatic Medicine, Chinese Academy of Medical Sciences, Chengdu, 610072, China

(6) Kyung Hee University, 26 Kyungheedae-ro, Dongdaemun-gu, Seoul 02447, Korea

(7) Department of Psychiatry, Medical University of Plovdiv, Plovdiv, Bulgaria

(8) Research Institute, Medical University Plovdiv, Plovdiv, Bulgaria

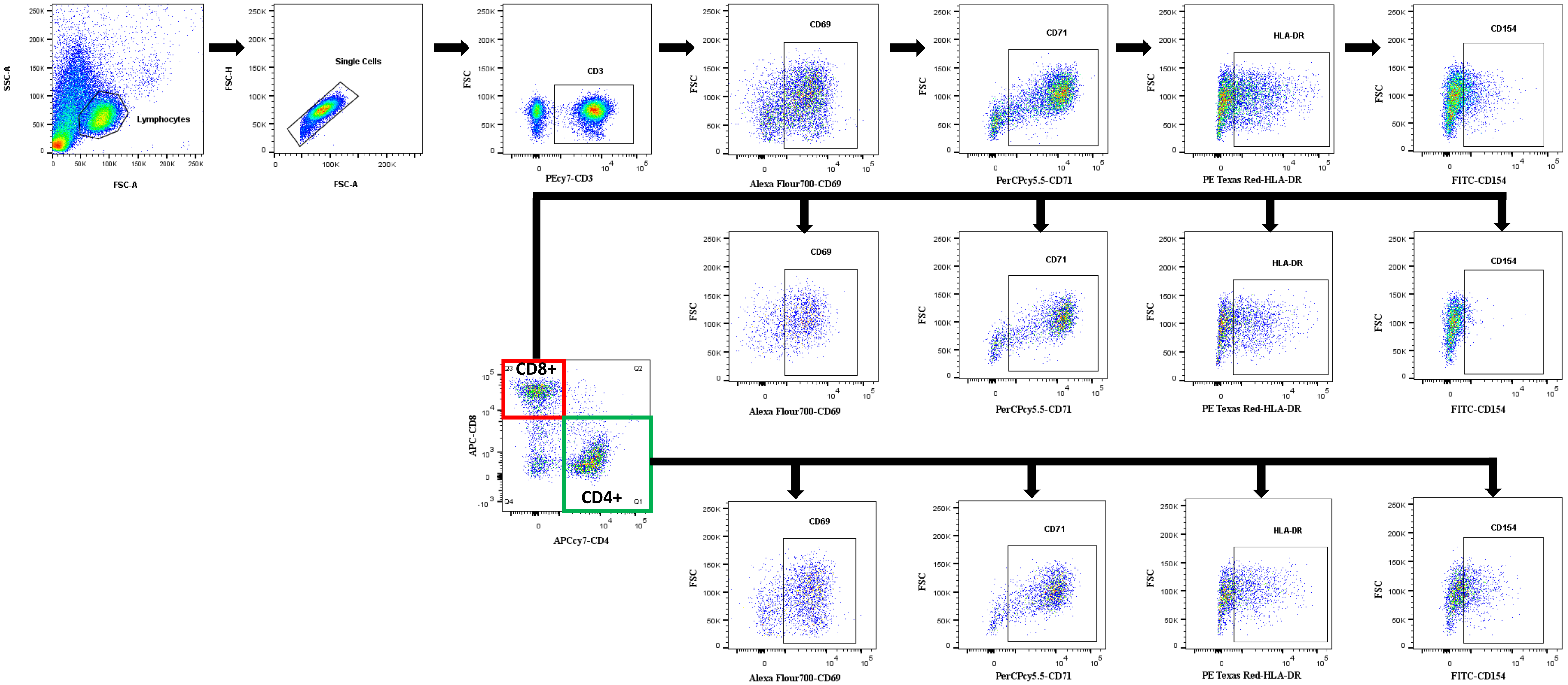


ESF1, Figure 1. Gating strategy to assess CD69, CD71, CD40L and HLA-DR -bearing CD3+, CD4+ and CD8+ cells

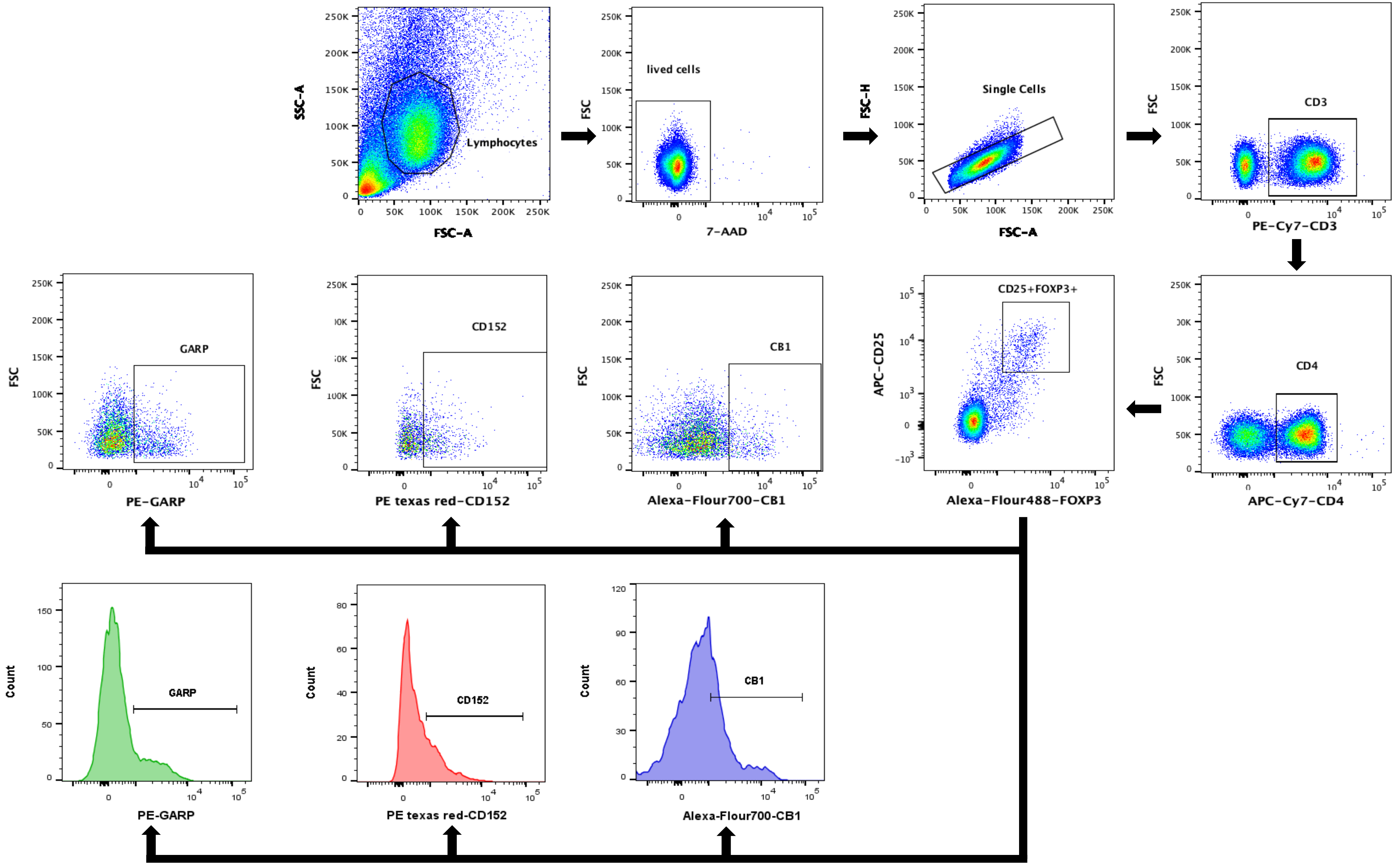


ESF1, Figure 2. Gating strategy to assess CB1, GARP and CD152 -bearing CD3+CD4+ CD25+FoxP3+ cells
