# Supplementary material for: T cell activation and deficits in T regulatory cells are associated with major depressive disorder and severity of depression
Source: Sci Rep. 2024 May 16;14:11177. doi: 10.1038/s41598-024-61865-y (PMC11096341; doi:10.1038/s41598-024-61865-y)
Supplement: Supplementary file 2 — Supplementary Tables. [file 41598_2024_61865_MOESM2_ESM.pdf]

ELECTRONIC SUPPLEMENTARY FILE 2 (ESF2)

T cell activation and deficits in T regulatory cells are associated with major depressive disorder and severity of depression

(1) Muanpetch Rachayon; (1,2) Ketsupar Jirakran; (3) Pimpayao Sodsai; (1) Atapol Sughondhabirom, (1,4-7) Michael Maes.

(1) Department of Psychiatry, Faculty of Medicine, Chulalongkorn University and King Chulalongkorn Memorial Hospital, the Thai Red Cross Society, Bangkok, Thailand

(2) Center of Excellence for Maximizing Children's Developmental Potential, Department of Pediatrics, Faculty of Medicine, Chulalongkorn University, Bangkok, Thailand

(3) Center of Excellence in Immunology and Immune-Mediated Diseases, Department of Immunology, Faculty of Medicine, Chulalongkorn University and King Chulalongkorn Memorial Hospital, Bangkok, Thailand

(4) Sichuan Provincial Center for Mental Health, Sichuan Provincial People's Hospital, School of Medicine, University of Electronic Science and Technology of China, Chengdu 610072, China

(5) Key Laboratory of Psychosomatic Medicine, Chinese Academy of Medical Sciences, Chengdu, 610072, China

(6) Kyung Hee University, 26 Kyungheedae-ro, Dongdaemun-gu, Seoul 02447, Korea

(7) Department of Psychiatry, Medical University of Plovdiv, Plovdiv, Bulgaria

(8) Research Institute, Medical University Plovdiv, Plovdiv, Bulgaria

ESF2, Table 1. **T**he different CD and cell surface markers, as well as their key functions, measured in the present study.

CD Marker	Alternative names and functions
CD3+	T3, pan T-cell marker
CD4+	T4, T-cell marker, T helper cell
CD8+	Leu2, T-cell marker, T cytotoxic cell
CD69+	Very early activation T-cell marker of especially tissue-resident immune cells (marker of tissue retention), involved in lymphocyte proliferation
CD71+	Transferrin receptor, early activation T cell marker, promotes T cell proliferation
CD40L or CD154	CD40 ligand, expressed on activated T cells, member of the TNF family, binds to CD40 (on CD8+ T cells, macrophages, antigen presenting cells, and B cells
CD3 + CD4 + CD69+	CD69+ co-expressing CD3 CD4 cells
CD3 + CD8 + CD69+	CD69+ co-expressing CD3 CD8 cells
CD3 + CD4 + CD71+	Proliferating CD3 + CD4+ cells
CD3 + CD8 + CD71+	Proliferating CD3 + CD8+ cells
CD3 + CD4 + CD40L+	Activated CD3 + CD4+ T cells
CD3 + CD8 + CD40L+	Activated CD3 + CD4+ T cells
CD25+FoxP3+CB1+ (gated from CD3+CD4)	CB1 bearing T regulatory (Treg) cells
CD25+FoxP3+CD152+ (gated from CD3+CD4)	CTLA-4 or CD152 is expressed on Treg cells, immune checkpoint that negatively regulates the immune response, CD152 bearing Treg cells

CD25+FOXP3+GARP+ (gated from CD3+CD4+)	Glycoprotein A repetitions predominant, TGF-β1 receptor, Treg activation marker, these Tregs release TGF-β1, promote immune tolerance

ESF2, Table 2. Cytokine and chemokines examined in the current study.

Protein abbreviations	Gene Symbol	Protein name	OOR samples (%)
IL-1β	IL1B	Interleukin-1β	0
IL-1RA	IL1RN	Interleukin-1 receptor antagonist	0
IL-2	IL2	Interleukin-2	0
IL-4	IL4	Interleukin-4	0
IL-6	IL6	Interleukin-6	0
CXCL8 / IL-8	CXCL8	C-X-C motif chemokine ligand 8	0
IL-9	IL9	Interleukin-9	0
IL-12	IL12	Interleukin-12	1
IL-15	IL15	Interleukin-15	0
IL-17	IL17	Interleukin-17	0
CCL11	CCL11	Eotaxin	0
G-CSF	CSF3	Granulocyte Colony Stimulating Factor, Colony Stimulating Factor 3	0
GM-CSF	CSF2	Granulocyte-macrophage colony-stimulating factor, Colony-stimulating factor 2	0
IFN-γ	IFNG	Interferon-γ	0
CXCL10 /IP-10	CXCL10	C-X-C motif chemokine ligand 8, Interferon gamma-induced protein 10	0
CCL2 / MCP1	CCL2	C-C Motif Chemokine Ligand 2	0
CCL3 / MIP-1α	CCL3	Macrophage inflammatory protein-1 alpha, C-C Motif Chemokine Ligand 3	0
CCL5 /RANTES	CCL5	C-C Motif Chemokine Ligand 5, Regulated Upon Activation, Normally T-Expressed, And Presumably Secreted	0
TNF-α	TNF	Tumor Necrosis Factor-Alpha	0

OOR: out of range (lower than the sensitivity of the assay). The results indicate that all analytes were measurable in all participants.

ESF2, Table 3. Description of the immune profiles used in this study.

Immune Profile	Members
**M1 macrophage**	IL-1β, sIL-1RA, IL-6, TNF-α, CXCL8, CCL3
**T helper-1**	IL-2, IFN-γ, IL-12
**IRS**	IL-1β, IL-6, TNF-α, CXCL8, CCL3, IL-2, IFN-γ, IL-12, IL-17, IL-15, G-CSF, GM-CSF, CXCL10, CCL5, CCL2
**T cell growth**	IL4, IL9, IL12, GM-CSF, IL-15
**Neurotoxicity**	IL-1β, IL-6, TNF-α, CXCL8, CCL3, IL-2, IFN-γ, IL-12, IL-17, CXCL10, CCL11, CCL5, CCL2

IRS: immune-inflammatory response system

ESF2, Table 4. Measurements of the frequencies of T cell activation markers in baseline (UNSTIM) and stimulated (STIM) conditions in major depressive disorder (MDD) and controls (HC).

Estimates

				95% Wald Confidence Interval	
MDD	UN_STIM	Mean	Std. Error	Lower	Upper
HC	UNSTIM	4.8226	1.59616	1.6942	7.9510
	STIM	50.4121	.96969	48.5115	52.3126
MDD	UNSTIM	6.2336	1.54829	3.1990	9.2682
	STIM	52.5532	1.21500	50.1719	54.9346

Results are shown as estimated marginal mean (in percentage) and SE.

ESF2, Table 5. Differences in unstimulated (UNST) and stimulated (STIM) changes of median immune fluorescence (MFI) of T lymphocytes in healthy controls (HC) and major depressed patients (MDD)

	**Condition**	**Diagnosis**		**Tests of Model effects**		
**MFI (z scores)**		**HC [a]** **n=20**	**MDD [c]** **n=28**	**Effects**	**Wald** **df=1**	**p**
CD3+CD69+	UNST	-0.665 (0.105)	-0.785 (0.2)	Group (G)	0.047	0.829
	STIM	0.702 (0.06)	0.761 (0.063)	Gxtime (T)	0.779	0.371
**CD3+CD71+**	UNST	-1.109(0.12) [c]	-0.791(0.088) [a]	G	4.222	**0.040**
	STIM	0.895(0.037)	0.931(0.035)	GXT	4.024	**0.045**
CD3+CD154+	UNST	-1.001 (0.098)	-0.889 (0.068)	G	0.336	0.562
	STIM	0.952 (0.049)	0.921 (0.06)	GXT	0.98	0.332
CD3+HLADR+	UNST	-0.992 (0.131)	-0.761 (0.131)	G	3.774	0.052
	STIM	0.807 (0.07)	0.882 (0.049)	GXT	0.613	0.434
CD4+CD69+	UNST	-0.843 (0.110)	-0.886 (0.127)	G	0.266	0.606
	STIM	0.776 (0.064)	0.929 (0.06)	GXT	1.469	0.225
**CD4+CD71+**	UNST	-1.109 (0.116) [c]	-0.79 (0.085) [a]	G	3.88	**0.049**
	STIM	0.928 (0.039)	0.908 (0.045)	GXT	6.144	0.013
CD4+CD154+	UNST	-1.013 (0.095)	-0.889 (0.063)	G	0.153	0.695
	STIM	0.981 (0.05)	0.909 (0.058)	GXT	1.882	0.170
**CD4+HLADR+**	UNST	-1.13 (0.138) [c]	-0.633 (0.119) [a]	G	8.42	**0.004**
	STIM	0.754 (0.078)	0.879 (0.077)	GXT	3.973	0**.046**

CD8+CD69+	UNST	-0.71 (0.293)	-0.56 (0.143)	G	0.027	0.870
	STIM	0.667 (0.086)	0.587 (0.092)	GXT	0.861	0.353
CD8+CD71+	UNST	-0.955 (0.093)	-0.928 (0.064)	G	0.807	0.369
	STIM	0.867 (0.065)	0.986 (0.05)	GXT	0.648	0.421
CD8+CD154+	UNST	-0.907 (0.099)	-0.94 (0.068)	G	1.017	0.313
	STIM	0.82 (0.057)	0.997 (0.066)	GXT	1.874	0.171
CD8+HLADR+	UNST	-0.919(0.128)	-0.855(0.116)	G	0.148	0.700
	STIM	0.873(0.062)	0.886(0.048)	GXT	0.081	0.766
CD25+ FOXP3+CB1+	UNST	-0.025 (0.160)	-0.376 (0.108)	G	1.047	0.306
	STIM	0.343 (0.29)	0.168 (0.214)	GXT	0.452	0.501
CD25+ FOXP3+CD152+	UNST	-0.43 (0.235)	-0.817 (0.171)	G	1.865	0.172
	STIM	0.728 (0.095)	0.622 (0.059)	GXT	1.229	0.268
CD25+ FOXP3+GARP+ %	UNST	-0.717(0.279)	-0.742(0.112)	G	0.052	0.820
	STIM	0.669(0.074)	0.773(0.057)	GXT	0.218	0.640

ESF2, Table 6. Measurements of the median fluorescence intensity of T regulatory cells in baseline (UNST) and stimulated (STIM) conditions in major depressive disorder (MDD) and healthy controls (HC).

Estimates

				95% Wald Confidence Interval	
Group	UNST_STIM	Mean	Std. Error	Lower	Upper
HC	UNST	-.1969023	.09951962	-.3919572	-.0018475
	STIM	.3848233	.16977562	.0520692	.7175774
MDD	UNST	-.4500016	.05696057	-.5616423	-.3383610
	STIM	.3268810	.10627718	.1185816	.5351805

Results are shown as estimated marginal mean (in percentage) and SE.

ESF, Table 7. Differences in unstimulated (UNST) and lipopolysaccharide + phytohaemagglutinin-stimulated (STIM) changes in various immune profiles in healthy controls (HC) and major depressed patients (MDD).

Variables (z scores)		HC n=20	MDD n=30	p
M1	UNST	-0.878 (0.061)	-0.851 (0.045)	
	STIM	0.607 (0.040)	1.032 (0.143)	0.004
Th-1	UNST	-1.385 (0.074)	-1.514 (0.051)	
	STIM	0.222 (0.085)	0.547 (0.152	0.063
IRS	UNST	-1.522 (0.096)	-1.528 (0.073)	
	STIM	0.123 (0.050)	0.615 (0.050)	0.002
T cell growth	UNST	-1.471 (0.092)	-1.439 (0.097)	
	STIM	0.033 (0.048)	0.541 (0.164)	0.003
Neurotoxicity	UNST	-1.615 (0.102)	-1.685 (0.064)	
	STIM	0.266 (0.065)	0.598 (0.125)	0.018

Results of GEE analyses with the 5 immune profiles as dependent variables and time (unstimulated versus stimulated), group (depression versus controls) and time by group interactions as explanatory variables. All results are shown as estimated marginal means (SE). Age, sex, body mass index, and tobacco use were not significant in these analyses. All the interaction patterns were significant ($p<0.01$), and all stimulated immune profiles were significantly higher than the unstimulated profiles ($p<0.001$). Pairwise comparisons show no significant differences in the unstimulated immune profiles between MDD and controls. The p values in this table indicate the pairwise comparisons of the stimulated profiles between MDD and controls. For immune profiles, see ESF, Table 2 and 3 for explanation.
